# Atomic reconfiguration among tri-state transition at ferroelectric/antiferroelectric phase boundaries in Pb(Zr,Ti)O_3_

**DOI:** 10.1038/s41467-022-29079-w

**Published:** 2022-03-16

**Authors:** Zhengqian Fu, Xuefeng Chen, Henchang Nie, Yanyu Liu, Jiawang Hong, Tengfei Hu, Ziyi Yu, Zhenqin Li, Linlin Zhang, Heliang Yao, Yuanhua Xia, Zhipeng Gao, Zheyi An, Nan Zhang, Fei Cao, Henghui Cai, Chaobin Zeng, Genshui Wang, Xianlin Dong, Fangfang Xu

**Affiliations:** 1 State Key Laboratory of High Performance Ceramics and Superfine Microstructures & Key Lab of Inorganic Functional Materials and Devices, Shanghai Institute of Ceramics, Chinese Academy of Sciences, Shanghai, China; 2 School of Aerospace Engineering, Beijing Institute of Technology, Beijing, China; 3 Institute of Nuclear Physics and Chemistry, China Academy of Engineering Physics, Mianyang, China; 4 Institute of Fluid Physics, China Academy of Engineering Physics, Mianyang, China; 5 Electronic Materials Research Laboratory, Key Laboratory of the Ministry of Education and International Center for Dielectric Research, School of Electronic and Information Engineering, Xi’an Jiaotong University, Xi’an, China; 6 Center of Materials Science and Optoelectronics Engineering, University of Chinese Academy of Sciences, Beijing, China; 7 Hitachi High-Tech (Shanghai) Co., Ltd., Shanghai, China; 8 School of Physical Science and Technology, ShanghaiTech University, Shanghai, China

**Keywords:** Ferroelectrics and multiferroics, Phase transitions and critical phenomena

## Abstract

Phase boundary provides a fertile ground for exploring emergent phenomena and understanding order parameters couplings in condensed-matter physics. In Pb(Zr_1-x_Ti_x_)O_3_, there are two types of composition-dependent phase boundary with both technological and scientific importance, i.e. morphotropic phase boundary (MPB) separating polar regimes into different symmetry and ferroelectric/antiferroelectric (FE/AFE) phase boundary dividing polar and antipolar dipole configurations. In contrast with extensive studies on MPB, FE/AFE phase boundary is far less explored. Here, we apply atomic-scale imaging and Rietveld refinement to directly demonstrate the intermediate phase at FE/AFE phase boundary exhibits a rare multipolar Pb-cations ordering, i.e. coexistence of antipolar or polar displacement, which manifests itself in both periodically gradient lattice spacing and anomalous initial hysteresis loop. In-situ electron/neutron diffraction reveals that the same parent intermediate phase can transform into either FE or AFE state depending on suppression of antipolar or polar displacement, coupling with the evolution of long-/short-range oxygen octahedra tilts. First-principle calculations further show that the transition between AFE and FE phase can occur in a low-energy pathway via the intermediate phase. These findings enrich the structural understanding of FE/AFE phase boundary in perovskite oxides.

## Abstract (web summary)

Ferroelectric/antiferroelectric phase boundary is both technologically and scientifically important. Here, the authors reveal the structure of intermediate phase involved in classical Pb(Zr_1-x_Ti_x_)O_3_.

## Introduction

Pb(Zr_1-x_Ti_x_)O_3_-based ceramics are one of the most famous perovskite oxides for its versatile engineering applications including piezoelectric, electrooptic, pyroelectric and antiferroelectric^1,2^. These properties have their composition between two technologically and scientifically important phase boundaries^3^. The first one separates polar regimes into tetragonal and rhombohedral symmetry, known as morphotropic phase boundary (MPB), which has become a key design principle for high performance lead-free and lead-based piezoceramics^4–7^. The second is ferroelectric/antiferroelectric (FE/AFE) phase boundary, where the dipoles ordering state changes abruptly from parallel to antiparallel arrangement. The phase transition at FE/AFE phase boundary can undergo significant electric, thermal or mechanical response by applying the conjugate fields. As a result, the FE/AFE phase boundary has also become a key principle for developing explosive energy conversion, electrocaloric refrigeration and high-power capacitors^8–11^.

Despite the fundamentally different nature, it is generally believed that excellent properties at MPB or FE/AFE phase boundary in Pb(Zr_1-x_Ti_x_)O_3_ arise from the extremely close energies of competing component phases, which makes order parameters be very sensitive to external stimulus^12–16^. By considering the diversity of structural instabilities, several significant advances in elucidating MPB effect have been made based on monoclinic phase, adaptive domain and so on^17–20^. Analogously, the frustrated domain structure and low-symmetry phase have been proposed for FE/AFE phase boundary^3,21^.

**Fig. 1:**
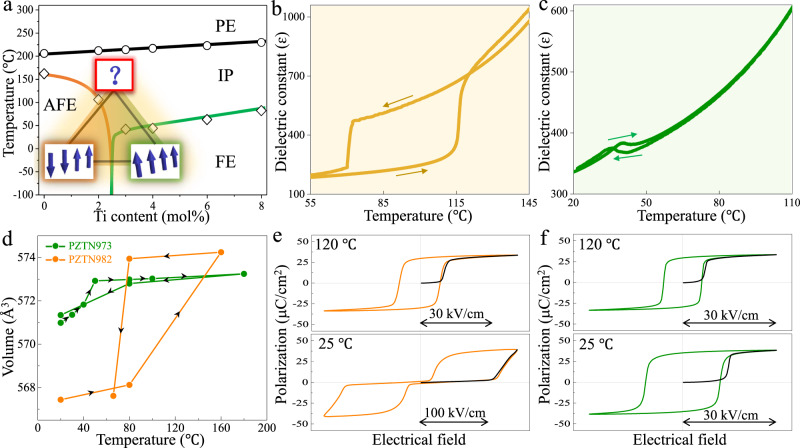
Macroscopic electric measurements. **a** Phase diagram of Pb_0.99_(Zr_1-x_Ti_x_)_0.98_Nb_0.02_O_3_ solid solution in composition range from *x* = 0 to *x* = 0.08. The PE, IP, AFE, and FE refer to paraelectric, intermediate, antiferroelectric, and ferroelectric phases, respectively. The blue arrows highlight the different arrangement of dipoles in AFE and FE phases. **b**, **c** The variations of dielectric constant for increasing and subsequently decreasing temperature spanning the phase transitions of IP-AFE and IP-FE for PZTN98/2 and PZTN97/3, respectively. **d** Lattice volumes (2a_p_ × 2 b_p_ × 2c_p_) of PZTN98/2 and PZTN97/3 as a function of temperature. **e**, **f** Temperature-dependent hysteresis loops for PZTN98/2 and PZTN97/3, respectively. The black curves indicate the polarization response for initial state.

Generally, there are three phases, i.e. AFE, intermediate-phase (IP) and FE phase exist at FE/AFE phase boundary and the tri-state can transform from one to another by changing temperature or composition (Fig. 1a). Among these phases, the IP is the most elusive because its structure was first assigned as rhombohedral symmetry without oxygen octahedra tilt while now is considered to be an incommensurately modulated structure in terms of antiphase boundary (APB) or antiphase domain (APD)^22–24^. However, for a long time, much of what we know about the IP is only limited to its morphology. The core information about how atoms shift in APB and APD remains unclear. As a result, it is unknown why the same parent IP could transform to either AFE or FE state, and how the electric dipoles configuration changes abruptly between antiparallel and parallel ordering across the FE/AFE boundary.

The biggest obstacle to clarifying the structural distortion in IP is the M-type superlattice reflections with characteristic 1/2{*ooe*} indices, which were experimentally observed many years ago, but also aroused fierce controversies including the generation mechanism of M-points, the correlation length of M-points associated ordered regions and the inconsistent data from electron and neutron diffraction^3,25–27^. There have been three popular mechanisms, i.e. chemical (A-site vacancy or B-site cations) ordering, oxygen octahedra tilt and antiparallel atomic displacement, that were proposed for generating M-type reflections. Many researchers have recognized the chemical ordering should be excluded since M-type reflections disappear on cooling, but there is little agreement with respect to the rest of two mechanisms. The confusion is mainly caused by the structural/microstructural characteristics of IP, i.e. the overall nanosized APBs and APDs rather than a long-range ordered single crystal structure. While traditional diffraction techniques were widely used for the attempt to decode the M-points, these structural determinations only result in average structure. Thus, the precise structure could only be resolved by atomic imaging as a successful example has been available in the structural determination of relaxor ferroelectrics^28^.

In this work, the Pb(Zr_1-x_Ti_x_)O_3_ ceramics with adding of 1 wt% Nb_2_O_5_ were prepared by solid-state reaction sintering in the composition range of x = 0–0.08. According to the temperature dependence of dielectric constant (Fig. S1), the regions of different phases are roughly plotted in Fig. 1a, which is consistent with those derived from Jaffe et al.^1^ and Noheda et al.^29^. We mainly focused on Pb_0.99_(Zr_0.98_Ti_0.02_)_0.98_Nb_0.02_O_3_ (PZTN98/2) and Pb_0.99_(Zr_0.97_Ti_0.03_)_0.98_Nb_0.02_O_3_ (PZTN97/3) ceramics because their composition is just located at the left and right side of FE/AFE phase boundary, respectively. By applying electrical measurements, in-situ neutron/electron diffraction, atomic-scale imaging and first-principles calculation, we achieve comprehensive understandings of FE/AFE phase boundary including anomalous first-loop, couplings of structural instabilities and mechanisms of phase transition.

## Results and discussion

To get insight into the physical nature of IP, AFE, and FE phases near phase boundary, we investigated the temperature-dependent dielectric and polarization responses. It is interesting to find that IP-AFE and IP-FE phase transitions have large and small thermal hysteresis (Fig. 1b, c), suggesting their strong and weak first-order characteristic, respectively. The calculated lattice volumes as a function of temperature (Fig. 1d), obtained from structural refinements against neutron powder diffraction data, also present a sharp jump of volume during IP-AFE transition for PZTN98/2 while a gradual change in IP-FE transition for PZTN97/3.

Figure 1e, f shows the polarization-electric field (P-E) hysteresis loops for IP, AFE, and FE phases. Clearly, the AFE phase matches well with the common belief for its double loops while the IP and FE phases achieve firm FE state after poling because the remanent polarization nearly equals with saturation polarization. Their first quarter of initial P-E loop (abbr. first-loop) is outside the subsequent loops, while ferroelectrics with typical domain switching behavior usually exhibit an inside first-loop^30^. The present outside first-loop implies that higher activation field is required for making the local structure to grow and to form long-range ordering. The local structure should refer to nanosized APB/APD in IP^22–24^ and monoclinic nanoregions in FE^31^. Moreover, it can be seen that the polarization of first-loop shows a slow increase with electric field rather than a sharp rise, which indicates the initial state in IP should not be considered as a metastable AFE phase. Please note that the similar anomalous first-loop has also been observed in the classical PMN relaxor^32^, which should be resulted from chemical and structural inhomogeneities. Thus, the measurements of first-loop may provide an easy and fast way to roughly judge whether the material has a complex structure deviated from typical FE phase.

**Fig. 2:**
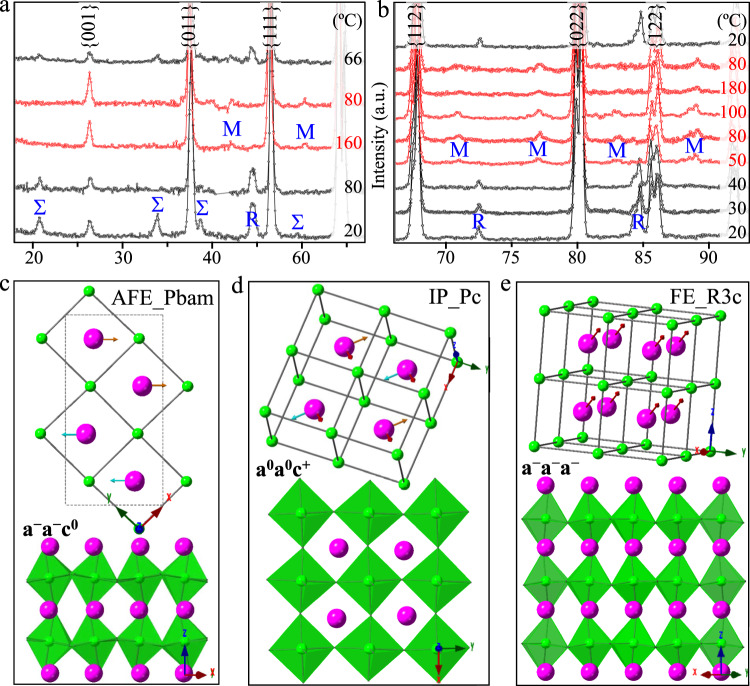
Average structural investigations. **a**, **b** In-situ neutron diffraction patterns of temperature-induced phase transitions of IP-AFE in PZTN98/2 and IP-FE in PZTN97/3, respectively. The patterns of IP are presented in red color. The Σ-type represents quadruple superlattice reflections while the R-type and M-type represent double superlattice reflections with characteristic indices of 1/2{*ooo*} and 1/2{*ooe*}, respectively. **c**‒**e** The behavior of Pb-cations displacement and oxygen octahedra tilts in AFE, IP and FE phase, respectively. The structural models are drawn based on the Rietveld refinement using Pc (taken at 80 °C of PZTN97/3), Pbam (taken at 20 °C), and R3c (taken at 20 °C). The projections of octahedra tilts in IP, AFE and FE phases are along [001]_p_, [1$\bar{1}$0]_p_ and [110]_p_ directions, respectively. The x, y, and z directions refer to pseudocubic cell.

Figure 2a, b shows the in-situ neutron diffraction patterns of PZTN98/2 and PZTN97/3, respectively, measured at increasing and subsequently decreasing temperatures (from bottom to top). The phase transitions of IP-AFE in PZTN98/2 and IP-FE in PZTN97/3 are explicitly evidenced by the evolution of their characteristic superlattice reflections. That is, the AFE phase has both Σ-type and R-type peaks, while the IP and FE phase only exhibit M-type and R-type peaks, respectively (also see peak indexing in Fig. S2). To clarify the source of these superlattice reflections, Rietveld refinements were carried out using full-profile data. It is found that the IP, AFE and FE phase can be well described by monoclinic symmetry with *Pc* space group, orthorhombic symmetry with *Pbam* space group and rhombohedral symmetry with *R3c* space group, respectively. Fig. S3 gives the corresponding Rietveld refinement patterns as well as transfer matrix between refined structure model and pseudocubic cell. For convenience, all crystallographic directions and planes refer to the pseudocubic unit cell in this study. The comparison of IP in PZTN98/2 and PZTN97/3 indicates that it has nearly the same structural distortions (Fig. S4), which agrees well with its similar anomalous electric behaviors.

According to the refined atomic coordinates (Table S1), the AFE phase (Fig. 2c) is featured by antiparallel Pb [1$\bar{1}$0]_p_-displacement (Σ_Pb_-type, ↑↑↓↓) and antiphase octahedra tilt (R-type, *a*^*-*^*a*^*-*^*c*^*0*^) while the FE phase (Fig. 2e) is characterized by parallel Pb [111]_p_-displacement (Г-type, ↑↑↑↑) and antiphase octahedra tilt (R-type, *a*^*-*^*a*^*-*^*a*^*-*^). Interestingly, the experimentally observed structural instabilities in IP (Fig. 2d) consist of parallel Pb displacement component along [uuv]_p_ directions (Г-type, ↑↑↑↑), antiparallel Pb [1$\bar{1}$0]_p_-displacement component (M_Pb_-type, ↑↓↑↓) and inphase octahedra tilt (M_O_-type, *a*^*0*^*a*^*0*^*c*^*+*^). Thus, it can be concluded that both M_Pb_-type and M_O_-type distortions contribute to the previously observed 1/2{*ooe*} superlattice reflections. In addition, the refined results of IP show that both the magnitude of M_Pb_-type displacement (5–10 pm) and M_O_-type tilt (3–4°) are small while the magnitude of Г-type displacement (15–30 pm) is relatively large.

To further confirm and understand the structural distortions in IP, real-space investigations were carried out by transmission electron microscopy (TEM). The dark-field image (Fig. S5) using the M-point shows the representative microstructure of IP, i.e. spontaneously assembled antiphase boundaries (APBs) which are periodically aligned normal to the <111> _p_ direction and segment the antiphase domains (APDs) with width of 5–10 nm. Also, we investigated the evolution of IP at changing temperature for both PZTN98/2 and PZTN97/3. It is found that the APDs in both compositions become wider with increasing temperature and PZTN98/2 has narrower APD than PZTN97/3 at 150 °C (Fig. S6). Atomic-scale high-angle annular dark-field (HAADF) and annular bright-field (ABF) images were acquired along both the [001]_p_ and [110]_p_ zone axis. According to our previous work^22^, the former direction is suitable for identifying the tilt system of oxygen octahedra while the latter can provide an edge-on observation of APBs. The positions of atomic columns are extracted using picometer-precision fitting^33^ (see the details in Methods).

**Fig. 3:**
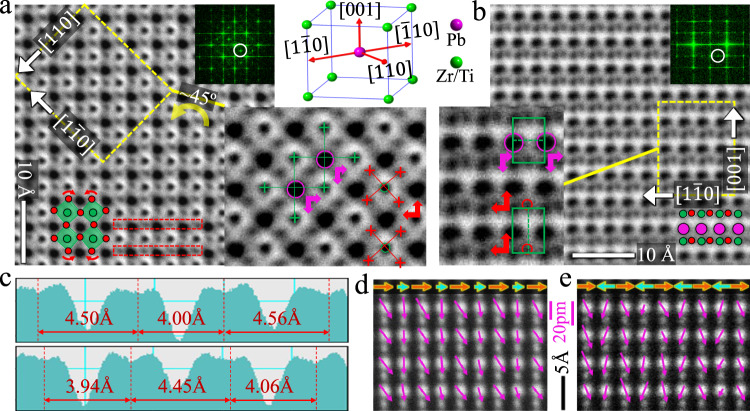
Atomic-scale characterization within antiphase domain in PZTN97/3. **a**, **b** The atom-resolved ABF images acquired within antiphase domain along [001]_p_ and [110]_p_ direction, respectively, where their fast Fourier transforms (FFTs) are inserted in upper-right corner. Their FFTs clearly show the M-points as marked by white circles. The magnified images of yellow boxes directly visualize the displacement of Pb (magenta) and oxygen octahedra (red) relative to average position of Zr/Ti (green) atomic positions. The illustration locating between **a** and **b** acts as a guideline for easily referring to lattice vectors. **c** Measurements of O-O distance derived from red rectangle boxes in **a** showing tilts of oxygen octahedra. **d**, **e** Two types of Pb modulated displacement (magenta arrows). The displacement vectors are overlaid on atomic-resolved HAADF images along [110]_p_ direction, where horizontal components associated with M-type superlattice reflection are highlighted with orange and cyan arrows.

Figure 3a and b are atom-resolved ABF images acquired within an APD along the [001]_p_ and [110]_p_ directions, respectively. Because Zr/Ti cations generally have very small displacements in PZT-based materials, their positions are averaged as reference sites for calculating Pb and O displacement. The following structural information can be extracted: (1) oxygen octahedra present inphase tilt along the [001]_p_ direction, which can be verified by superposing corner-linked octahedra onto the image (Fig. 3a). This tilt system can be further identified by measuring O-O distances across Pb-cations (Fig. 3c), where neighboring horizontal O-Pb-O atomic rows have staggered arrangement of different O–O distances; (2) besides inphase tilts, oxygen octahedra are obviously deviated away from the equilibrium position and move in the opposite direction with Pb-cations (see red and magenta arrows). (3) Pb-cations exhibit displacement components along all of [001]_p_, [110]_p_ and [1$\bar{1}$0]_p_ directions. The [001]_p_- and [110]_p_-components are common parallel displacement and can be composite into [uuv]_p_-displacement (Г-type, ↑↑↑↑). The [1$\bar{1}$0]_p_-components of adjacent Pb columns are also parallel but modulated with one large and one small value (M_Pb_-type, ↑**↑**↑**↑**, see the displacement vectors mapping in Fig. S7).

Based on atomic-scale investigations of several APDs, it is found that Pb [1$\bar{1}$0]_p_ displacement does not necessarily exhibit parallel M_Pb_-type (↑**↑**↑**↑**) only but can also present antiparallel M_Pb_-type (↑↓↑↓). For clarity, M_Pb_-type (↑**↑**↑**↑**) and M_Pb_-type (↑↓↑↓) will be called polar and antipolar modulated displacement, respectively, for comparing with common Г-type (↑↑↑↑) displacement. The two types of modulations are represented by Pb displacement maps in Fig. 3d, e, where the horizontal components associated with M-type superlattice reflection are highlighted with orange and cyan arrows.

**Fig. 4:**
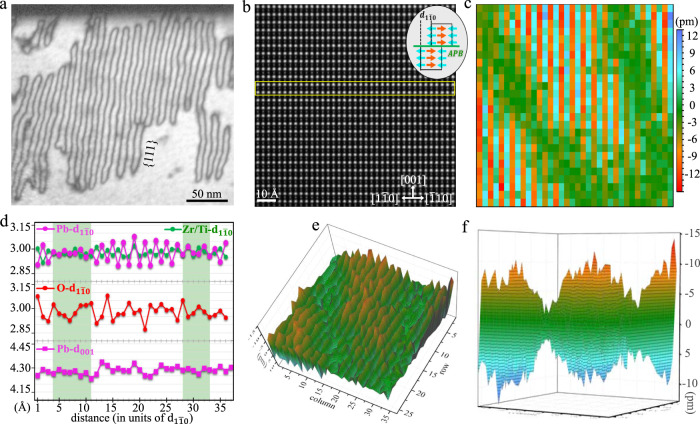
Antiphase boundaries driven lattice oscillation in PZTN97/3. **a** Spontaneously assembled APBs with long-period ordering. A large-area view of whole grain is presented in Fig. S5. **b**, **c** The atom-resolved HAADF image viewed along [110]_p_ direction and corresponding two-dimensional Pb horizontal displacement mapping, respectively, where the APB manifest itself as a U-like green curve. The inset in **b** illustrates an ideal model of APB based on antipolar displacement. **d** Quantified profiles obtained from yellow rectangle box in **b** show variations of lattice spacing across APBs, which are marked with green background. The O-${{{{{{\rm{d}}}}}}}_{1\bar{1}0}$ profile is calculated from corresponding ABF image in Fig. S8. **e**, **f** The 3D views Pb-displacement maps. The projected direction in **f** is nearly parallel with antiphase boundary.

To investigate the IP in terms of periodic APBs, we acquired relatively low magnified atomic-scale HAADF image (Fig. 4b). By mapping Pb horizontal displacement (Fig. 4c), it can be clearly seen that the APB manifests itself as a U-like green curve with a width of about 2 nm (also see the Pb-${{{{{{\rm{d}}}}}}}_{1\bar{1}0}$ map in Fig. S8), which agrees well with the loop morphology in Fig. 4a. The Pb-cations again exhibit antipolar modulated displacement along the [$1\bar{1}0$]_p_ direction in APDs as evidenced by alternate red and cyan color. Thus, periodic APBs can be modeled by mutual shift of a ${{{{{{\rm{d}}}}}}}_{1\bar{1}0}$ spacing between adjacent domains, as schematically illustrated in the inset of Fig. 4b. The lattice spacing profiles (Fig. 4d) measured from the yellow box in Fig. 4b indicate that all Pb-, Zr/Ti- and O-${{{{{{\rm{d}}}}}}}_{1\bar{1}0}$ are modulated in an alternately large and small way. In contrast to Pb-${{{{{{\rm{d}}}}}}}_{1\bar{1}0}$, the Zr/Ti-${{{{{{\rm{d}}}}}}}_{1\bar{1}0}$ modulation is very small and O-${{{{{{\rm{d}}}}}}}_{1\bar{1}0}$ modulation is less ordered. The fact that Pb-d_001_ in APD and at APB do not present a significant difference implying that the in-plane modulated displacements along the [1$\bar{1}$0]_p_ direction are not strongly coupled with the out-plane Г-type displacement along the [001]_p_ direction. Interestingly, oscillation of Pb-${{{{{{\rm{d}}}}}}}_{1\bar{1}0}$ shows gradient change in magnitude across APB, which leads to the deduction that APBs tend to be ferroelectric because they lose antipolar displacement (see the color bar for green APB in Fig. 4c). The 3D views Pb-displacement maps in Fig. 4e, f provide better visualization to show the gradient change of lattice spacing across antiphase boundaries.

**Fig. 5:**
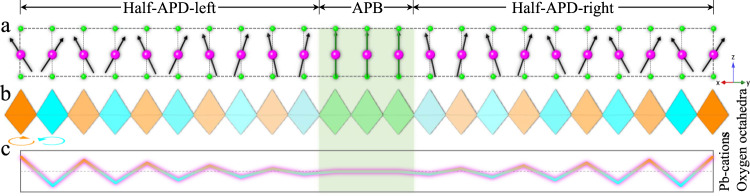
The ideal atomic representative model for a larger scale picture of IP state. **a** Pb displacements represented by arrows vary across an APB. Pb displacements without horizontal components in APB are highlighted by green arrows. **b** Oxygen octahedral tilts represented by gradation of color vary across an APB. Oxygen octahedral tilts with zero value in APB are highlighted by green colour. **c** A schematic profile illustrates combination of gradient oscillation of Pb horizontal displacements and oxygen octahedra tilts across an APB.

Figure 5 models an ideal atomic representative for a large-scale picture to make a further clearer description of IP state. In this model, the Pb displacements are coupled with oxygen octahedral tilts and the Zr/Ti displacements are assigned as zero because they are significantly smaller than Pb displacements. Figure 5a clearly shows that the horizontal component of Pb displacements gradually decreases towards an APB while the vertical component of Pb displacements maintains. Figure 5b presents that the tilt angle of oxygen octahedra, which is represented by gradation of color, also decreases gradually across the APB. Figure 5c further illustrates combination of gradient oscillation of Pb horizontal displacements and oxygen octahedra tilts via a schematic profile. It can be seen that the average structure by simple superposition of APB and APD still exhibits inphase octahedra tilt and coexistence of parallel and antiparallel Pb displacements, which is consistent with the one derived from neutron diffraction. It should be noted that the alternating arrangement of antiphase boundary and antiphase domain involves lattice spacing exhibiting periodical gradient oscillation in the whole crystal, which cannot be defined as a long-range ordered single crystal with a determined symmetry or space group.

After clarifying the structural characteristics of IP, the tri-state transition among AFE, IP and FE can be comprehensively understood from three aspects.

**Fig. 6:**
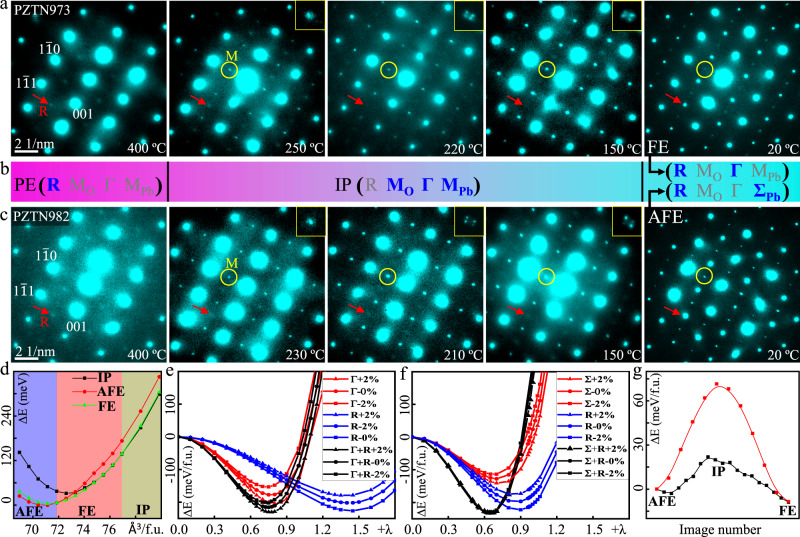
Phase transition mechanism. **a**, **c** Evolution of SAED patterns during decreasing temperature for PZTN98/2 and PZTN97/3, respectively. The M-type and R-type superlattice reflections are marked by yellow circles and red arrows, respectively. The M-points in IP are magnified and inserted in upper-right corners. **b** Evolution of structural instabilities. The symbols of blue color refer to dominant structural instabilities while those of gray color represent the very small or disappeared structural instabilities. **d** The energy difference as function of volume for AFE, FE and IP phases, where the minimum for AFE phase is set as 0 eV. **e**, **f** The frozen-phonon potential of soft modes driving PE into FE or AFE state, respectively, under different strains. **g** Comparison of two possible pathways for phase transition between AFE and FE by using NEB method.

Firstly, temperature-induced IP-FE/AFE transition. Figure 6a, c shows the evolution of crystal structure investigated by in-situ selected-area electron diffraction (SAED) along <110 > _p_ zone axes. The SAED patterns were acquired with long exposure time for visualizing weak reflections, which can hardly be observed in neutron diffraction. Above T_c_, both PZTN97/3 and PZTN98/2 exhibit R-type (see SAED patterns at 400 °C in Fig. 6) and M-type (Fig. S9) diffuse superlattice reflections. The M-points are obviously weaker than the R-points. Thus, temperature-induced phase transition for FE/AFE phase boundary does not start from ideal cubic symmetry. Instead, the PE phase exhibits complex local ordered structures, which is mainly associated with antiphase oxygen octahedra tilts. When temperature decreases just below the Curie point, both PZTN97/3 (250 °C) and PZTN98/2 (230 °C) immediately exhibit sharp M-points. Upon further decreasing temperature by 20–30 °C, the M-points start to split suggesting formation of periodic APBs in IP. The fact that the non-split M-points are just maintained in such a narrow temperature window indicates the long-range ordered (meaning in whole grain) coexistence of M_Pb_-type and Г-type displacements is metastable and periodic APB arrays will spontaneously develop to stabilize their competition. Interestingly, diffuse R-points always exist in the whole IP. When further cooling to near room temperature (20 °C), the diffuse R-points ultimately evolve into sharp state for both PZTN97/3 and PZTN98/2. This phenomenon indicates that phase transition of IP-FE and IP-AFE have order-disorder character. It should be noted that the systematic absences of Σ-points make only M-points keep in AFE phase. The M-points in FE phase become diffuse again and finally disappear at about −50 °C (Fig. S9). For convenience, the evolution of different structural instabilities during phase transition induced by temperature is listed in Fig. 6b. It can be seen that (1) M_O_ distortion is strengthened by suppressing R distortion in PE-IP phase transition and R distortion is strengthened by suppressing M_O_ distortion in IP-FE/IP-AFE transition, indicating the competition among different oxygen octahedra tilt systems; (2) the similar multipolar displacements in IP ultimately evolve into Г and Σ distortion in FE and AFE phase, respectively, implying the competition among different Pb displacement orderings; (3) R&Г/R&Σ distortions synchronously become stronger in IP-FE/IP-AFE transition, showing the cooperation between oxygen octahedral tilts and Pb displacement orderings. The observed couplings of structural instabilities during phase transition in PZTN98/2 should provide important experimental evidence for exploring origin of antiferroelectricity in doped-PbZrO_3_^34,35^.

Secondly, composition-induced FE↔AFE phase transition at room temperature. The volume difference about 0.6% between AFE and FE phase implies “volume” may have important influence on the phase structure (Fig. 1d). For simplification, we performed first-principles calculations of PbZrO_3_ with different volume to simulate this composition induced volume effect. Figure 6d shows AFE is the most stable state with smallest volume and then FE state tends to be stable as the volume increases followed by the IP when the volume further increases. Microscopically, Fig. 6e, f show the frozen-phonon double-well potential of soft modes driving PE into FE and AFE phase, respectively, under different strains. It can be seen that all soft modes induce double well potential, but R induces deeper energy barrier (*E*_H_) than Г/Σ. Moreover, the coupling of R&Г for PE-FE transition (R&Σ for PE-AFE transition) shows even deeper *E*_H_, indicating the transitions are actually induced by two coupled soft modes. Interestingly, the *E*_H_ at Γ and Σ get deeper (shallower) under tensile (compressive) strain while the *E*_H_ at R shows the opposite trend. For example, with 2% tensile strain, the relative changes of the *E*_H_ at R driving the FE and AFE are very close (22.73 vs 22.54 meV), however, the relative change of *E*_H_ at Γ driving FE transition is much larger than *E*_H_ at Σ driving AFE transition (−26.45 vs −13.48 meV), as shown in Table S2a. Therefore, under 2% tensile strain, the net energy gain (${E}_{H}^{{{{{{\rm{N}}}}}}}$) of R&Г modes and R&Σ modes induces −3.72 meV lower and 9.06 meV higher than zero strain state, respectively. Analogously, under 2% compressive strain, ${E}_{H}^{{{{{{\rm{N}}}}}}}$ of R&Г modes and R&Σ modes are −0.49 meV and −11.47 meV, respectively. Such a phenomenon suggests FE state is favorable with tensile strain while AFE phase is favorable with compressive strain, which is consistent with our experimental results, i.e. PZTN97/3 with larger volume possesses FE state while PZTN98/2 with smaller volume exhibits AFE state.

Thirdly, the crucial role of IP during FE↔AFE phase transition. We simulated pathways for FE ↔ AFE transition without and with IP included during nudged elastic band (NEB) calculation (Fig. 6g). It can be seen that the energy barrier is as large as 72 meV if the FE ↔ AFE transition occurs directly without IP. However, the energy barrier decreases dramatically to 15 meV if IP is introduced in the transition pathway, i.e., FE ↔ IP ↔ AFE. Therefore, by introducing IP, the phase transition between FE and AFE can be carried out along a low-energy pathway, realizing the process of otherwise extremely high energy involving sharp change between parallel and antiparallel polarization configurations. Moreover, our findings indicate that the IP can catalyze the transition process through a low-energy pathway is mainly attributed to its dual-nature structural distortions, i.e. common displacement components along [uuv]_p_ direction (Г-type, ↑↑↑↑) and modulated displacement components along [1$\bar{1}$0]_p_ direction (either M_Pb_-type, ↑**↑**↑**↑** or M_Pb_-type, ↑↓↑↓). This character highly resembles the MPB in PZT, where rhombohedral (*R*3*m*) phase can change to tetragonal (*P*4*mm*) phase via a bridge monoclinic phase (*Cm*), i.e., *R*3*m* ↔ *Cm* ↔ *P*4*mm*. Because the *Cm* allows an atomic displacement in (110)_p_ mirror plane, it can easily change to the one allowed in *R*3*m* or *P*4*mm*^15,31^. Thus, the IP can also be considered as a bridge phase because the direction of M_Pb_-type and Г-type displacement are allowed in AFE and FE phase, respectively.

In summary, we have achieved comprehensive understandings of FE/AFE phase boundary in PZT solid solutions. It is found that the IP phase plays an important role in the tri-state transition among AFE, IP, and FE phases. On the one hand, the multipolar character of IP makes its dipole ordering can evolve into either parallel (FE) or antiparallel (AFE) arrangement by decreasing temperature. In consideration of structural instabilities, IP-FE and IP-AFE transitions are mainly involved in suppressing M_Pb_-type distortions and Г-type distortions, respectively, which is accompanied by the evolution of oxygen octahedral tilts from inphase to antiphase state. On the other hand, the multipolar character of IP acts as a bridge between FE and AFE states, averting the sharp change between parallel and antiparallel arrangement by changing composition. The low-energy pathway with IP included in FE ↔ AFE transition implies that a similar multipolar intermediate phase may also occur in field-induced transition between FE and AFE state. These results render important implications for understand the intermediate or bridge phase at phase boundaries.

## Methods

### Materials synthesis

The ceramics were prepared through the conventional solid-state reaction method. The high-purity raw materials of Pb_3_O_4_ (99.26%), ZrO_2_ (99.87%), TiO_2_ (99.38%), and Nb_2_O_5_ (99.38%) were stoichiometrically weighed according to the nominal compositions, whereas 0.5 wt% of Pb_3_O_4_ excess amount was added in compensation for the volatilization of Pb during sintering. These raw materials were mixed and calcined at 850 °C for 2 h. After fine milling, the powders were pressed into disks of 16 mm diameter at 150 MPa. The pellets were then covered with powders of same composition, and sintered at ~ 1300 °C for 1 h in sealed crucibles. Finally, the sintering samples were cut into disks of various diameter and various thickness, and the silver electrodes were burned to be used to measure the electric properties.

### Electric properties characterization

The high-temperature dielectric spectra were obtained by an LCR meter (Model HP 4284 A, USA) with a furnace where the heating rate is 2 °C/min. The low-temperature dielectric spectra were measured using an impedance analyzer (Agilent E4980A) combined with a physical property measurement system (PPMS-9, Quantum Design). The circling dielectric properties with temperature for PZTN98/2 and PZTN97/3 ceramics were also measured by the LCR meter (Model HP 4284 A, USA) where the temperature was controlled by the oven (Vötsch VT7004, Germany) between 10 °C and 150 °C. The polarization versus electric field (*P*-*E*) hysteresis loops of PZTN98/2 and PZTN97/3 ceramics were measured with a ferroelectric measuring system (TF 2000 analyzer, aix ACCT Co., Germany). Before each polarization measurement at a temperature, the ceramics was heated to 450 °C for 30 min to recover a history-independent state, and was then cooled to the desired temperature for measurement.

### In-situ neutron diffraction and Rietveld refinements

Neutron diffraction experiments were carried out on a high-resolution neutron diffractometer at the Key Laboratory of Neutron Physics, Institute of Nuclear Physics and Chemistry, China Academy of Engineering Physics. The wavelength of neutron is 1.8846 Å. PZTN98/2 and PZTN97/3 ceramic plates were sealed in a TiZr can and put in a furnace. The scattering data were collected at different temperatures by covering the scattering angle 2*θ* of 15–160°. Rietveld refinements were carried out using software TOPAS-ACADEMIC^36^. During the refinements, the scale factor, background parameters, Pseudo-Voight peak-shape function, and absorption correction coefficient, as well as structural parameters were refined.

### Conventional transmission electron microscopy (CTEM)

The specimens were prepared by mechanical thinning and Ar+ ion-milling. The ion-milling voltage is gradually decreased from 3 keV to 0.5 keV to reduce ion-beam damage. Specimens were then coated with a thin layer of carbon to minimize charging under the electron beams. The SAED patterns were carried out on JEOL JEM-2100F microscope. The in-situ temperature field was applied by Gatan Model 652 double-tilt heating holder. It should be noted that the transition temperatures in CTEM could be slightly deviated from those in electric measurement. Nevertheless, they reveal the same phase transition sequences.

### Scanning transmission electron microscopy (STEM)

The atomic-scale high-angle annular dark-field (HAADF) and annular bright-field (ABF) images were carried out on Cs-corrected Hitachi HF5000 microscope. The microscope settings were: probe size in UHR mode and convergence semi-angle of 20 mrad and collection semi-angle of 60–320 mrad (HAADF) and 11–22 mrad (ABF). The images were acquired under conditions of fast scanning and cross-correlation summing of multiple frames to minimize sample drift. Please note that the data in both Fig. 3 and Fig. 4 were acquired for PZTN97/3 at room temperature while the electron-beam illumination should heat the sample to a certain extent. By comparing the Curie point in dielectric spectra (Fig. S1) and in-situ TEM (Fig. 5), heating due to the electron-beam is estimated to increase the temperature of the local illuminated sample area by 20–40 °C. Thus, although the environment temperature for TEM is at room temperature, the actual sample temperature (45–65 °C) just makes PZTN97/3 be in IP phase (appearing above ~40 °C). The fitting of atomic columns was done by MATLAB code, a least squares estimation algorithm for accurate and precise quantification of the atomic column positions and intensities with considering overlap between neighboring atomic columns^33^. The fitting of the atomic columns for Pb-cations, Zr/Ti-cations and O-anions in this work has a 95% confidence interval of about 1 pm, 2 pm, and 5 pm, respectively. The Pb displacement was calculated by methods as follows: (1) the Pb offset from the center of its four Zr/Ti neighbors in [001]_p_ projected ABF image; (2) the Pb offset from the center of its two vertical Zr/Ti neighbors in [110]_p_ projected ABF and HAADF image; (3) the horizontal components of Pb displacement based on deviation of Pb-${{{{{{\rm{d}}}}}}}_{1\bar{1}0}$(distance between two neighboring Pb atoms along horizontal direction) from the average ${{{{{{\rm{d}}}}}}}_{1\bar{1}0}$ spacing of one Pb atomic row in [110]_p_ projected HAADF image. Both calculation methods of average Zr/Ti center and average Pb-${{{{{{\rm{d}}}}}}}_{1\bar{1}0}$ deviation give similar displacement maps (Fig. S10).

### First-principle calculations

All calculations were performed via density functional theory (DFT) as implemented in the Vienna *ab* initio simulation package (VASP)^37^. In this work, electron-ion interactions were described as exchange and correlation potential of revised Perdew–Burke–Ernzerhof (PBE^38^). For relaxation of structures, the Brillouin zone was sampled by Monkhorst-Packgrid grip^39^ with k-spacing of 0.2 Å^−1^. The energy convergence criterion of 1.0 × 10^−5^ eV and the tolerance for force convergence of 0.01 eV/Å were used during structural relaxation via conjugate gradient minimization. In addition, we calculated the kinetic barriers by the climbing image nudged elastic band (CI-NEB) method^40^. The supercell of FE and IP with 40 atoms were built, with the same number of atoms as the AFE structure. The AFE structure was set as initial structure and FE as final state structure, for the AFE-FE transition calculation. The initial intermediate structures for transition state calculations were built from linearly interpolating between AFE and FE structures. Then the stable transition states and their total energies can be calculated by the NEB method. For the transition with IP structure, the similar NEB calculations were performed in the transitions between AFE-IP and IP-FE, as shown in Fig. 5g. The optimized lattice parameters of *Pbam*, *R*3c and *P*c can be found in Table S2b.

### Reporting summary

Further information on research design is available in the Nature Research Reporting Summary linked to this article.

## Supplementary information

Supporting Information

Reporting Summary

## Data Availability

The data that support the findings of this study are available from the corresponding author upon reasonable request.
