# Supplementary material for: Atomic reconfiguration among tri-state transition at ferroelectric/antiferroelectric phase boundaries in Pb(Zr,Ti)O3
Source: Nat Commun. 2022 Mar 16;13:1390. doi: 10.1038/s41467-022-29079-w (PMC8927586; doi:10.1038/s41467-022-29079-w)
Supplement: Supplementary file 1 — Supporting Information [file 41467_2022_29079_MOESM1_ESM.pdf]

Supplementary Information

Atomic reconfiguration among tri-state transition at ferroelectric/antiferroelectric phase boundaries in $Pb(Zr,Ti)O_3$

Zhengqian Fu, Xuefeng Chen, Henchang Nie, Yanyu Liu, Jiawang Hong, Tengfei Hu, Ziyi Yu, Zhenqin Li, Linlin Zhang, Heliang Yao, Yuanhua Xia, Zhipeng Gao, Zheyi An, Nan Zhang, Fei Cao, Henghui Cai, Chaobin Zeng, Genshui Wang, Xianlin Dong, Fangfang Xu

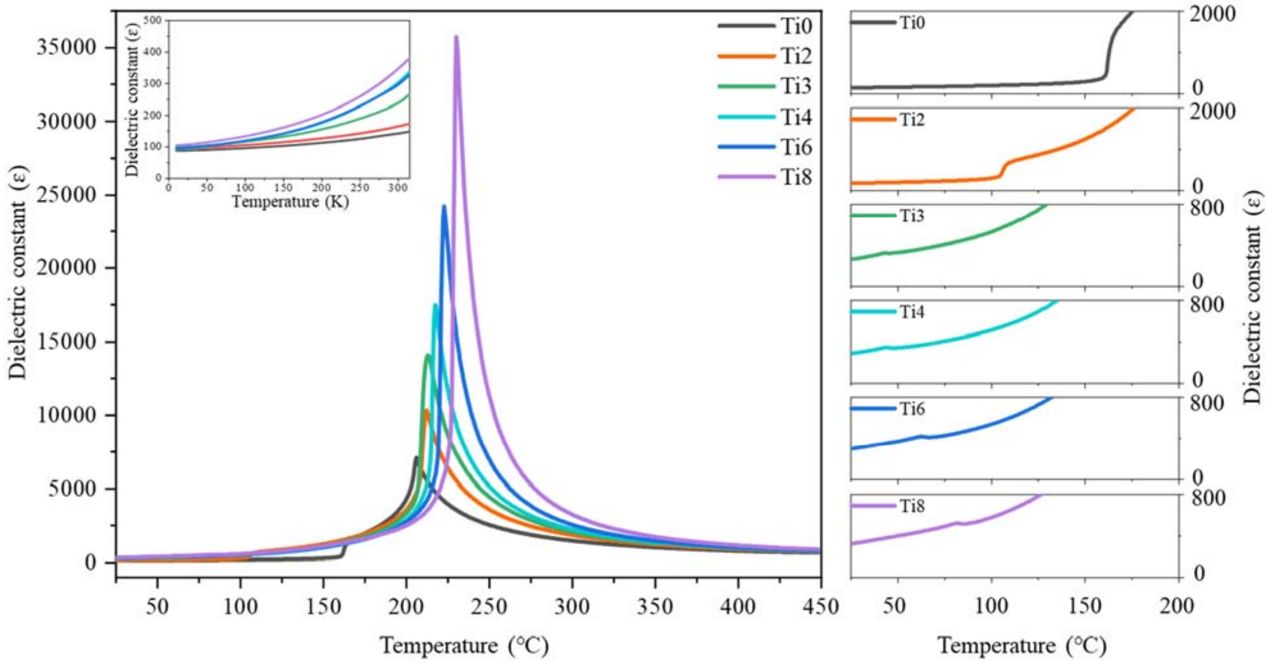

Fig. S1 Variations of dielectric constant during increasing temperature for $Pb_{0.99}(Zr_{1-x}Ti_x)_{0.98}Nb_{0.02}O_3$ solid solution. The Curie peaks can be clearly observed in left panel. The insert is low-temperature dielectric response where no phase transition is observed. The phase transitions of IP-AFE and IP-FE are clearly presented in right panel by magnifying dielectric response in the range between 25 ˚C and 200 ˚C.

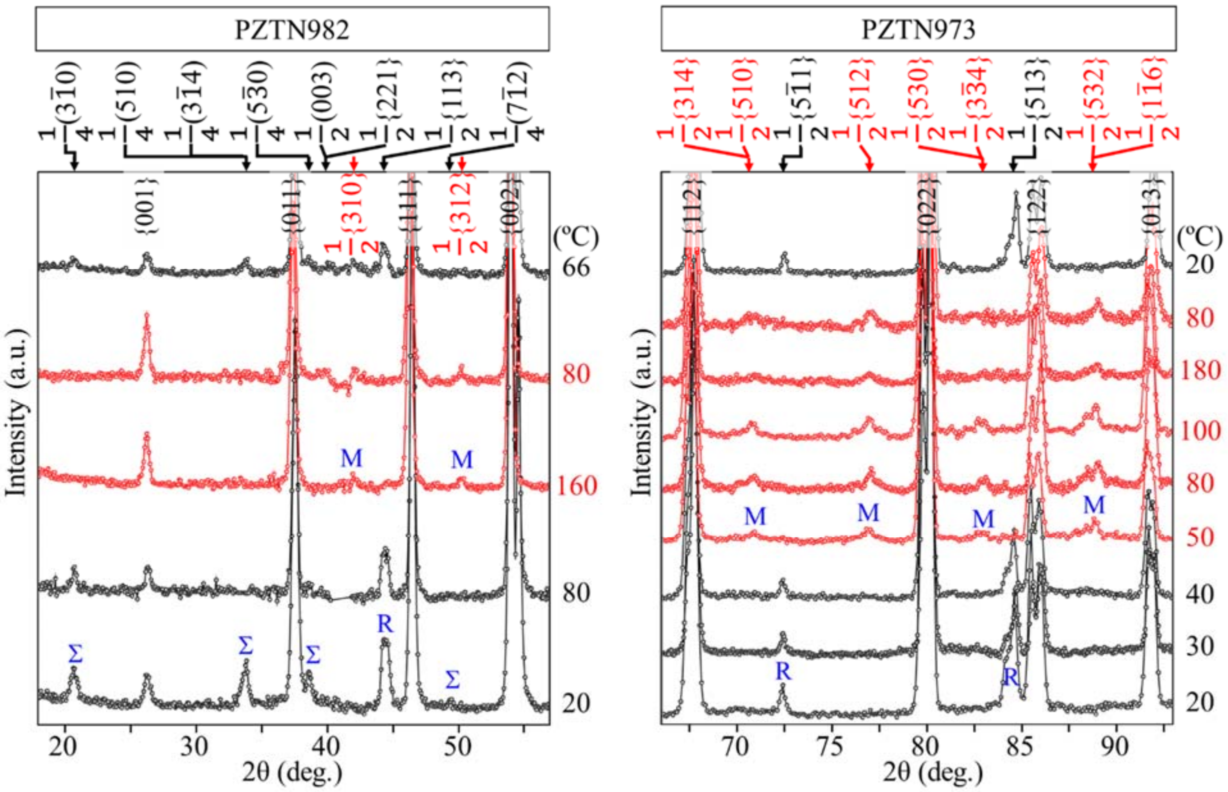

Fig. S2 Indexing superlattice reflections for in-situ neutron diffraction of PZTN98/2 and PZTN97/3. The Σ-type reflections represent quadruple superlattice while the R-type and M-type reflections represent double superlattice with characteristic indices of 1/2{*ooo*} and 1/2{*ooe*}, respectively. The "*o*" and "*e*" refer to odd and even indices, respectively.

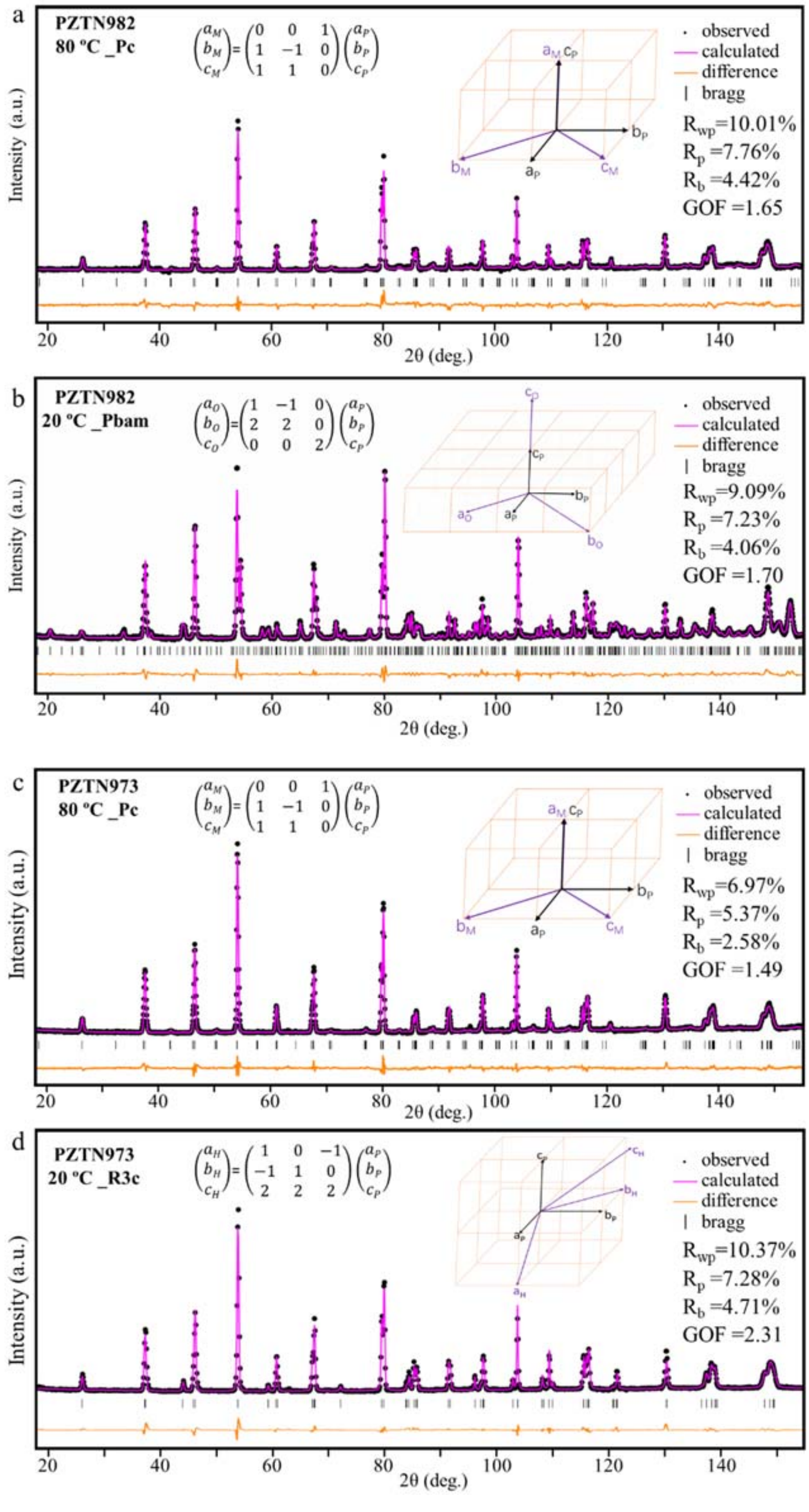

Fig. S3 (a, b) Rietveld fitting patterns of PZTN98/2 using *Pc* and *Pbam* model for IP (taken at 80 ºC) and AFE (taken at 20 ºC) phase, respectively. (c, d) Rietveld fitting patterns of PZTN97/3 using *Pc* and *R*3*c* model for IP (taken at 80 ºC) and FE (taken at 20 ºC) phase, respectively. The insets show relationship with corresponding transfer matrix between refined structure model and their pseudocubic cell, and reliability factors in Rietveld analysis of neutron diffraction data. The subscript "M", "O", "H" and "P" refers to monoclinic, orthorhombic, rhombohedral (hexagonal setting) and pseudocubic unit cell, respectively.

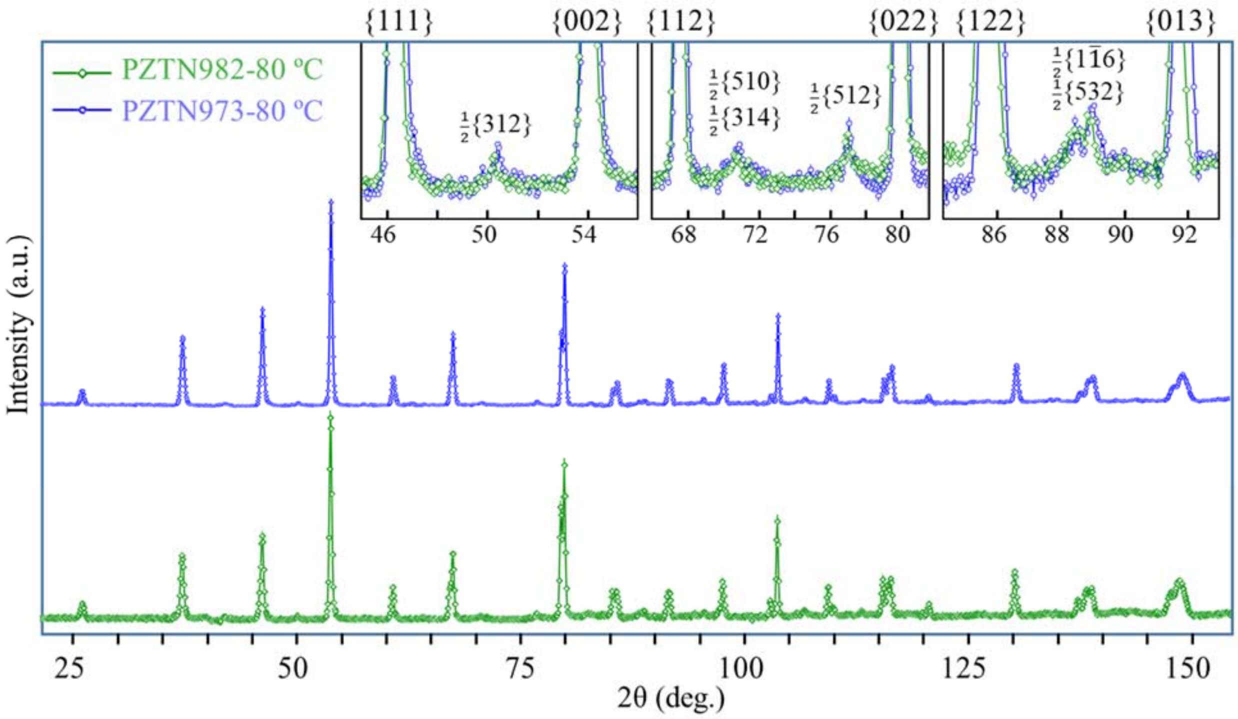

Fig. S4 The comparison of IP in PZTN98/2 and PZTN97/3 indicates that the IP phase has similar structural distortions. The insets show magnified M-type reflections with characteristic indices of 1/2{*ooe*}.

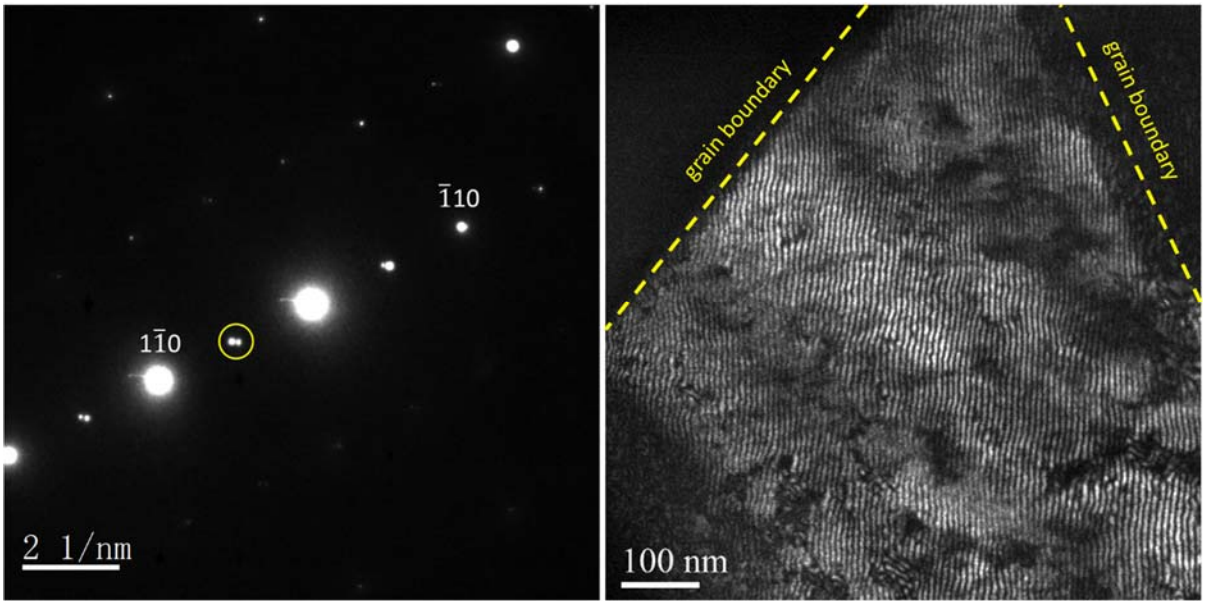

Fig. S5 Selected-area electron diffraction pattern and dark-field image for ordered APBs array in PZTN97/3. The tilted-beam condition is set for better contrast. The yellow circle marks M-type points used for dark-field image, where the whole grain with nearly parallel APBs can be seen clearly.

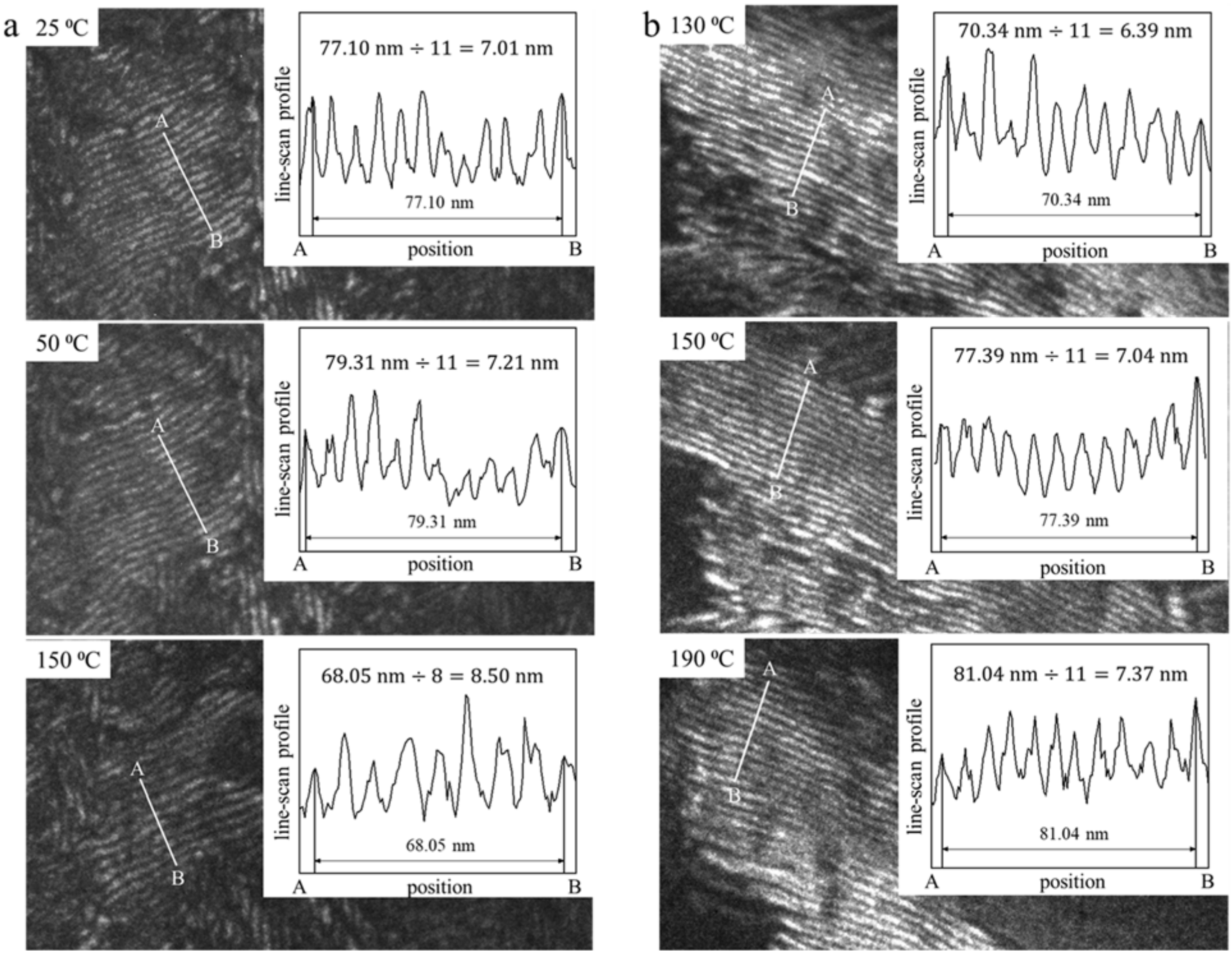

Fig. S6 (a) and (b) show evolution of the IP as temperature changing for PZTN97/3 and PZTN98/2, respectively. It can be seen that the APDs become wider with increasing temperature and the PZTN98/2 has narrower APD than PZTN97/3 at the same 150 °C.

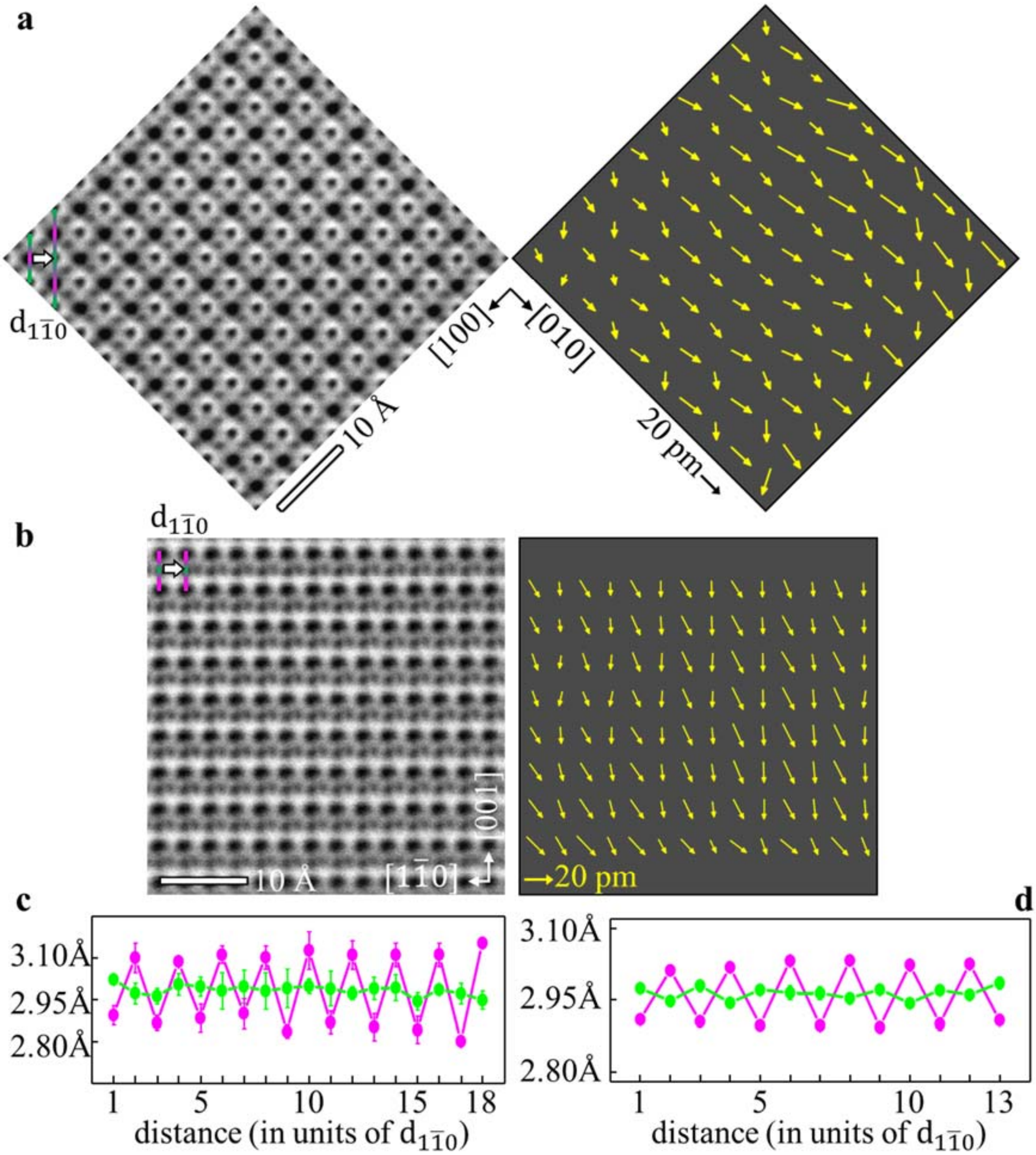

Fig. S7 (a, b) The high-resolution ABF image of Figs. 3a and 3b and their corresponding two-dimensional Pb displacement mapping. (a) is a counterclockwise rotation of 45° for Fig. 3a. It can be clearly seen that the horizontal components of Pb displacement exhibit modulation of one large and one small between neighboring columns. (c, d) The Pb-$d_{1\bar{1}0}$ (magenta) and Zr-$d_{1\bar{1}0}$ (green) profiles derived from ABF image in (a) and (b), respectively. The $d_{1\bar{1}0}$ lattice spacing is given by calculating the average horizontal distance of neighboring columns of Pb or Zr, which is illustrated by the vertical magenta/green lines in (a) and (b). The modulation of Pb-$d_{1\bar{1}0}$, one large and one small, is consistent with the modulation of Pb displacement.

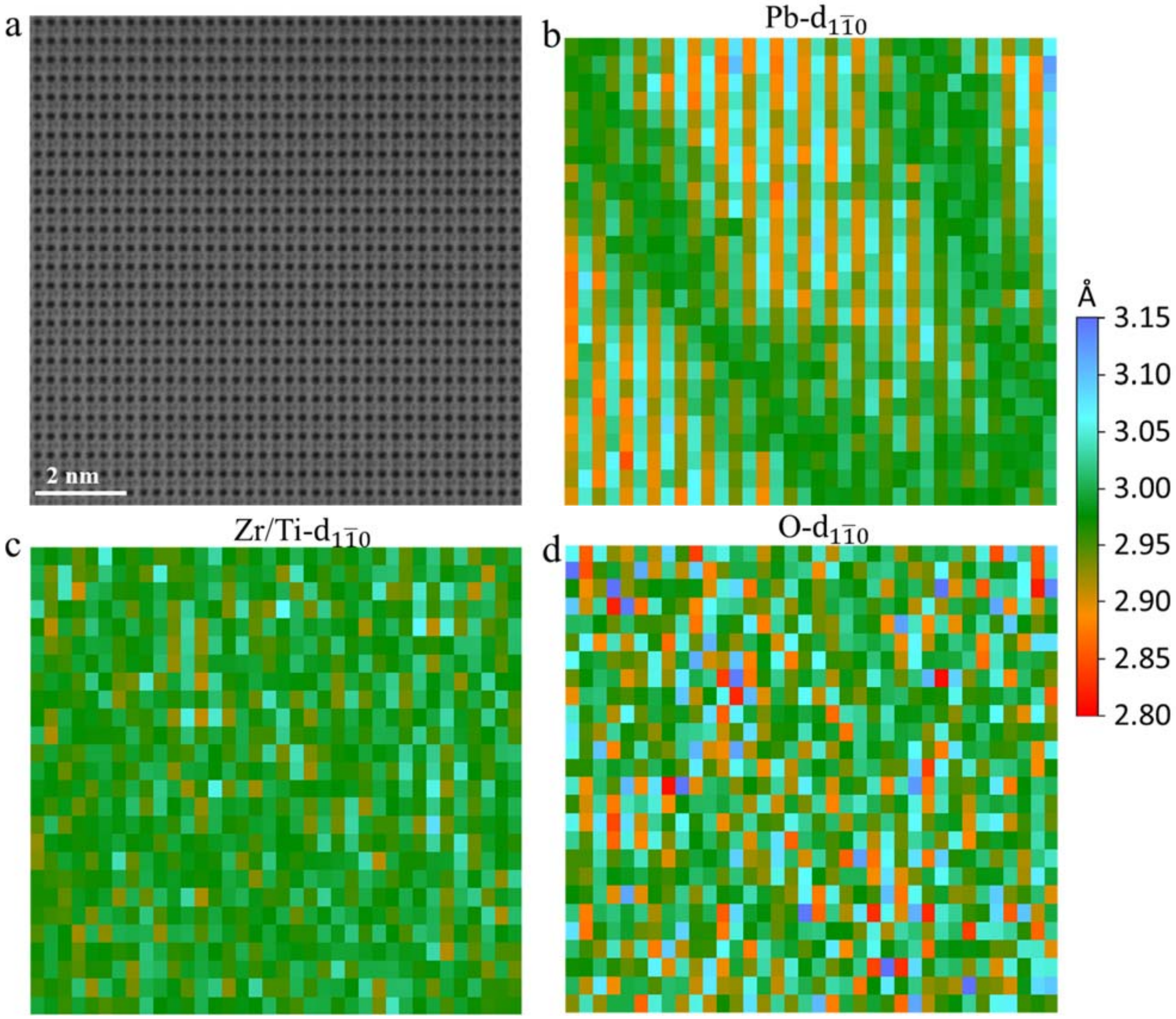

Fig. S8 (a~d) Two-dimensional lattice spacing maps of Pb-$d_{1\bar{1}0}$, Zr/Ti-$d_{1\bar{1}0}$ and O-$d_{1\bar{1}0}$ derived from the ABF image along $[110]_p$ direction. The ABF image is the same area with HAADF image in Fig. 3b.

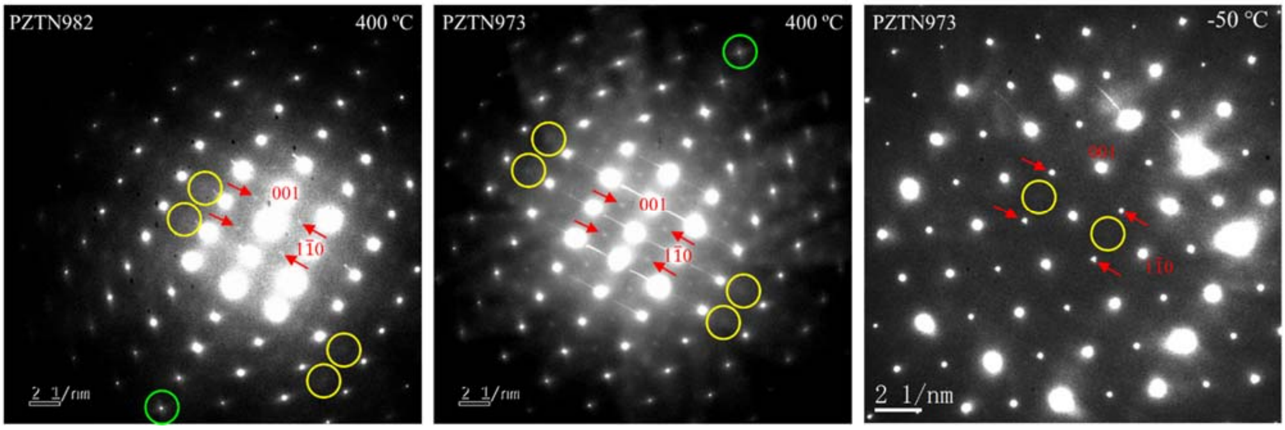

Fig. S9 The SAED patterns of PE phase at 400 °C and FE phase at -50 °C. In PE phase, it can be seen that both PZTN97/3 and PZTN98/2 exhibit R-type (red arrow) and M-type (yellow circle) diffuse superlattice reflections. The M-points are weaker than R-points. In FE phase at -50 °C, the M-type superlattice reflections disappear. The butterfly-shaped diffuse scattering (green circle) around basic reflections also shows existence of local order but its structural origins will not be discussed in this work. The bright tails of basic reflections are caused by long exposure time.

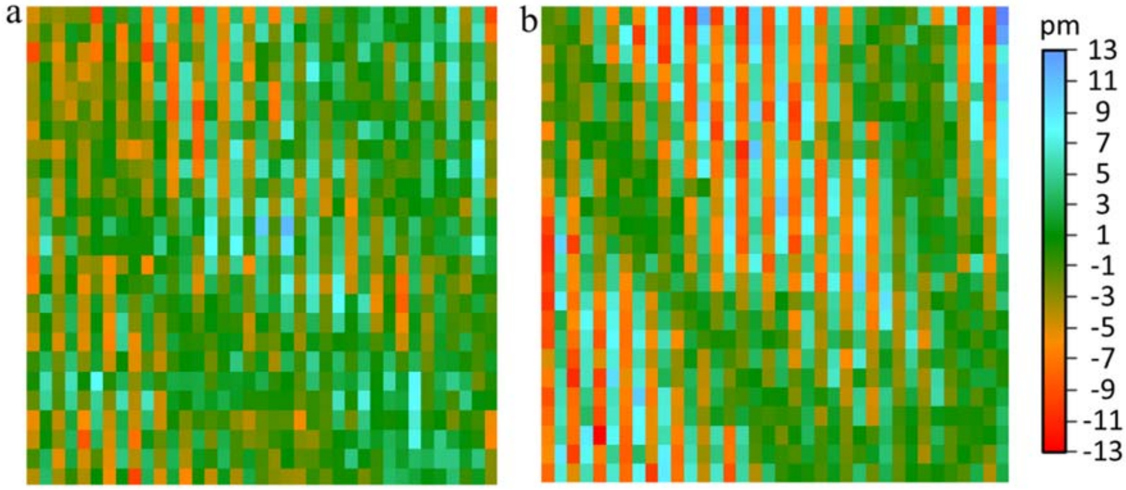

Fig. S10 (a) and (b) are Pb horizontal displacement derived from the HAADF image in Fig. 4b using different method. (a) Calculation of total Pb displacement by averaging Zr/Ti cations positions as reference center, i.e. the middle point of the vertical line connecting two neighboring Zr/Ti atom columns. (b) Calculation based on the deviation of Pb-$d_{1\bar{1}0}$(distance between two neighboring Pb atoms along horizontal direction) from the average $d_{1\bar{1}0}$ spacing of one row of Pb atom. The (a) is less ordered than (b) because the HAADF image is acquired at relatively low magnification and thus has less pixels for Zr/Ti atoms. Anyway, both calculation methods reveal the antipolar Pb displacement and antiphase boundary.

a

		**PZTN982_*Pc*_80 °C**			
α = γ = 90°	β = 89.683(6)°	*a* =4.1557(5)Å	*b* =5.8630(6) Å	*c* = 5.8892(5) Å	*V* = 143.49(2) $Å^3$
atom	Wyckoff positions	$x//[001]_p$	$y// [1\bar{1}0]_p$	$z//[110]_p$	B_iso
Pb1	2a	0.071(1)	0.758(2)	0.05(1)	3.1(2)
<B>	2a	0.543(1)	0.2492(9)	0.02(1)	0.6(1)
O1	2a	0.03(1)	0.279(2)	-0.03(1)	0.7(3)
O2	2a	0.546(5)	0.514(6)	0.259(4)	3.0(6)
O3	2a	0.5	0	0.25	2.5(5)

b

		**PZTN982_*Pbam*_20 °C**			
α = β = γ = 90°		*a* =5.8707(1)Å	*b* =11.7576(3)Å	*c* = 8.2203(1)Å	*V* = 567.41(2) $Å^3$
atom	Wyckoff positions	$x//[1\bar{1}0]_p$	$y// [110]_p$	$z//[001]_p$	B_iso
Pb1	4g	0.712(2)	0.1286(7)	0	1.86(9)
Pb2	4h	0.712(2)	0.1223(7)	0.5	1.86(9)
<B>	8i	0.2426(6)	0.1243(9)	0.251(1)	0.16(8)
O1	4g	0.290(2)	0.100(1)	0	0.7(2)
O2	4h	0.276(2)	0.155(1)	0.5	0.4(2)
O3	8i	0.035(1)	0.2627(5)	0.2164(7)	0.6(1)
O4	4f	0	0.5	0.279(1)	1.5(2)
O5	4e	0	0	0.261(2)	0.7(2)

c

		**PZTN973_*Pc*_80 °C**			
α = γ = 90°	β = 89.689(3)°	*a* =4.1541(2)Å	*b* =5.8599(3)Å	*c* = 5.8843(2)Å	*V* = 143.24(1) $Å^3$
atom	Wyckoff positions	$x//[001]_p$	$y// [1\bar{1}0]_p$	$z//[110]_p$	B_iso
Pb1	2a	0.032(8)	0.766(1)	0.020(7)	2.69(4)
<B>	2a	0.541(4)	0.2502(9)	0.008(4)	-0.14(7)
O1	2a	0.078(4)	0.262(2)	0.084(3)	2.8(2)
O2	2a	0.518(3)	0.487(3)	0.277(2)	-0.2(1)
O3	2a	0.5	0	0.25	2.5(2)

d

		**PZTN973_*R3c*_20 °C**			
α = β = 90°	γ= 120°	*a* =5.85450(9) Å	*b* =5.85450(9)Å	*c* = 14.4318(3)Å	*V* = 428.38(16)$Å^3$
atom	Wyckoff positions	$x//[10\bar{1}]_p$	$y// [\bar{1}10]_p$	$z//[111]_p$	B_iso
Pb1	6a	0	0	0.2830(3)	2.6(1)
<B>	6a	0	0	0.0129(4)	1.2(1)
O1	18b	0.2035(6)	0.3463(7)	0.0833(3)	2.3(1)

Table S1 (a, b) Atomic coordinates of PZTN98/2 obtained by full-profile Rietveld refinement using *Pc* and *Pbam* model for IP and AFE phase, respectively. (c, d) Atomic coordinates of PZTN97/3 obtained by full-profile Rietveld refinement using *Pc* and *R3c* model for IP and FE phase, respectively.

a

		2%	-2%
FE	E_H^{Γ}	-26.45	24.10
	E_H^{R}	22.73	-24.59
	E_H^{N}	-3.72	-0.49
AFE	E_H^{Σ}	-13.48	14.08
	E_H^{R}	22.54	-25.55
	E_H^{N}	9.06	-11.47

b

	a (Å)	*b* (Å)	*c* (Å)	*β*
Pbam	5.87	11.77	8.18	90.00°
*R*3c	5.84	5.84	14.42	120.00°
*P*c	4.17	5.87	5.93	89.20°

Table S2 (a) Energy barrier E_H of soft modes and their net value ($E_H^{N} = E_H^{\Gamma/\Sigma} + E_H^{R}$), driving PE into FE or AFE state, respectively, under different strains. (b) The optimized lattice parameters of *Pbam*, *R*3c and *P*c.
